# Secretion of BMP-2 by tumor-associated macrophages (TAM) promotes microcalcifications in breast cancer

**DOI:** 10.1186/s12885-021-09150-3

**Published:** 2022-01-04

**Authors:** Shuo Wang, Haiyang Jiang, Caiwei Zheng, Ming Gu, Xinyu Zheng

**Affiliations:** 1 Department of Breast Surgery, First Affiliated Hospital, China Medical University, 155 North Nanjing Street, Shenyang, 110001 Liaoning China; 2 University of Miami Miller School of Medicine, Miami, FL USA; 3 Lab 1, Cancer Institute, First Affiliated Hospital, China Medical University, Shenyang, Liaoning China

**Keywords:** Breast cancer, Tumor-associated macrophages (TAMs), Microcalcifications, BMP-2, CD163, CD68

## Abstract

**Introduction:**

Breast microcalcifications is a characteristic feature in diagnostic imaging and a prognostic factor of breast cancer. However, the underlying mechanisms of breast microcalcifications formation are not fully understood. Previous studies have shown that upregulation of bone morphogenetic protein 2 (BMP-2) is associated with the occurrence of microcalcifications and tumor-associated macrophages (TAMs) in the tumor microenvironment can secrete BMP-2. The aim of this study is to elucidate the role of secretion of BMP-2 by TAMs in promoting microcalcifications of breast cancer through immunohistochemical staining and co-culturing of breast cancer cells with TAMs.

**Methods:**

A total of 272 patients diagnosed with primary invasive breast cancer from January 2010 to January 2012 in the First Hospital of China Medical University were included in this study. Immunohistochemical staining of CD68 (marker of entire macrophages), CD168 (marker of the M2-like macrophages) and BMP-2 were performed on 4-μm tissue microarray (TMA) sections. Following induction, THP-1 cells were differentiated to M2-like TAMs and were then co-cultured with breast cancer cells (MCF-7). Calcifications and BMP-2 expression were analyzed by Alizarin Red S staining and western blot, respectively.

**Results:**

Immunohistochemical analysis showed that the expression of CD168 was significantly increased in tissues with microcalcifications and was correlated with the expression of BMP-2 and poor prognosis. The formation of cellular microcalcifications and BMP-2 expression were significantly increased in MCF-7 cells co-cultured with TAMs compared with MCF-7 cells alone.

**Conclusions:**

These findings support the hypothesis that TAMs secrete BMP-2 to induce microcalcifications in breast cancer cells and influence prognosis via multiple pathways including BMP-2 and its downstream factors.

**Supplementary Information:**

The online version contains supplementary material available at 10.1186/s12885-021-09150-3.

## Introduction

Breast microcalcifications are small deposits of calcium with a diameter of < 0.5 mm in breast tissues, and are well known as an important biomarker and mammographic feature of breast cancer, especially in the early detection of non-palpable breast cancer [1]. Though microcalcifications were found in approximately 55% of nonpalpable breast cancers [2], the underlying mechanism of their formation is still poorly understood. Currently, bone morphogenetic proteins 2 (BMP-2) has been implicated to play a role in the formation of microcalcifications. BMPs are growth factors of the TGF-β super family and BMP-2 is a specific and key regulator of osteoblast-like cells [3–5]. Studies have shown that BMP-2 can induce breast cancer cells to acquire osteoblastic characteristics, leading to the formation of microcalcifications [6]. A recent study also suggested that the active processes of microcalcifications are due to the osteoimmunological disorders [7].

Tumor-associated macrophages (TAMs) are one of the major types of tumor infiltrating immune cells in the extracellular environment [8] and were shown to accumulate around microcalcifications in breast cancer [9]. High TAMs levels are associated with poor prognosis and clinicopathologic features in many human tumors [10–14]. TAMs that are involved in breast cancer include a spectrum of phenotypes with M1-like and M2-like phenotypes as two extremes [15] and can either exhibit antitumor capacity (M1-like phenotype) or increase cancer cell growth (M2-like phenotype) [16]. Most TAMs have M2-like phenotype (CD163) [17] and breast cancer cells can secrete factors to promote macrophage differentiation toward the M2-like phenotype [18]. CD68, a pan-macrophage marker, can be used as an effective indicator for both M1 and M2 macrophages [19], whereas the CD168 is a scavenger receptor specific to the M2 macrophages [20].

Our studies, amongst others, have presented robust evidence that breast cancer with microcalcifications is associated with poor clinical outcome [5, 21–28] and BMP-2 is upregulated in tissues with microcalcifications [3–5]. Based on the information currently available in the literature, BMP-2 is believed to be mainly secreted by cells in the tumor microenvironment, but not the breast cancer tumor cells themselves [29]. TAMs, as an important component of the tumor microenvironment, were found to be able to secrete BMP-2 that contributes to vascular calcification [30]. Therefore, in this study, we quantified the TAMs levels in breast cancer tissue and determined its correlation with the expression of BMP-2 and microcalcifications by immunohistochemical evaluation. Further experiment demonstrated the role of secretion of BMP-2 by TAMs in promoting microcalcifications of breast cancer through co-culturing of breast cancer cells with TAMs.

## Materials and methods

### Patients and tissues

Our study cohort consisted of 272 patients with primary invasive breast cancer who were treated at the First Hospital of China Medical University from January 2010 to January 2012. Patients who were diagnosed with invasive breast cancer of stage I to III and received pre-surgery mammography were included. Patients who are younger than 20 years or older than 80 years were excluded. Patients with distant metastasis at the time of diagnosis, previous history of other malignant neoplasms including breast cancer, and those who were not candidates for radical surgery were also excluded. Patients with rare histologic subtypes and HER-2(2+) without fluorescence in in situ hybridization (FISH) test were also excluded.

Patients were followed for a median of 115 months (ranging from 105 to 125 months) after initial surgical treatment. Relevant clinical and pathological parameters were shown in Table 1. Archived formalin-fixed paraffin-embedded breast tissues were collected and were made into tissue microarray (TMA). All of the carcinomas were histologically verified as invasive breast cancer based on the criteria established by the World Health Organization and the molecular subtypes of breast carcinoma were also determined. This study was approved by the ethics committee of the First Affiliated Hospital (AF-SOP-07-1.1-01) (Shenyang, China).

**Table 1:** Patients’ clinicopathological parameters and correlation with TAMs

Parameters	High CD68 (%) ***N*** = 123	LOW CD68 (%) ***N*** = 149	χ2 value	***P*** value	High CD163 (%) ***N*** = 125	LOW CD163 (%) ***N*** = 147	χ2 value	***P*** value
**Age**			2.469	0.116			0.773	0.379
≤ 45	43 (35.0)	39 (26.2)			41 (32.8)	41 (27.9)		
>45	80 (65.0)	110 (73.8)			84 (67.2)	106 (72.1)		
**Tumor size**			0.925	0.819			2.522	0.471
T1	38 (30.9)	52 (34.9)			36 (28.8)	54 (36.7)		
T2	76 (61.8)	89 (59.7)			80 (64)	85 (57.8)		
T3	6 (4.9)	6 (4.0)			7 (5.6)	5 (3.4)		
T4	3 (2.4)	2 (1.3)			2 (1.6)	3 (2.0)		
**Axillary metastasis**			1.544	0.672			1.783	0.619
N0	58 (47.2)	79 (53.0)			62 (49.6)	75 (51.0)		
N1	33 (26.9)	35 (23.5)			28 (22.4)	40 (27.2)		
N2	20 (16.3)	25 (16.8)			24 (19.2)	21 (14.3)		
N3	12 (9.8)	10 (6.7)			11 (8.8)	11 (7.5)		
**Hormonal receptor**			2.177	0.140			3.622	0.057
Positive	88 (71.5)	94 (63.1)			91 (72.8)	91 (61.9)		
Negative	35 (28.5)	55 (36.9)			34 (27.2)	56 (38.1)		
**Her-2**			2.420	0.120			3.007	0.083
Positive	30 (24.4)	25 (16.8)			31 (24.8)	24 (16.3)		
Negative	93 (75.6)	124 (83.2)			94 (75.2)	123 (83.7)		
**Microcalcifications**			9.141	0.002			28.060	0.000
With microcalcifications	46 (37.4)	31 (20.8)			55 (44.0)	22 (15.0)		
Without microcalcifications	77 (62.6)	118 (79.2)			70 (56.0)	125 (85.0)		
**Follow up (month)**	116	115			115	115		
**Recurrence**	24	20			29	15		

*T* docetaxel, *P* platinum, *E* epirubicin, *C* cyclophosphamide, *F* 5-fluorouracil

### Immunohistochemical staining

Immunohistochemical examination was performed on TMA sections with a thickness of 4-μm. The staining of BMP-2 was performed as previously described [5]. For the staining of CD68 and CD163, antigen retrieval was first performed (EDTA, pH 9.0) after deparaffinization and blocking of the endogenous peroxidase. Sections were then incubated overnight at 4 °C with the primary rabbit anti-CD68 (ab125212; Abcam, Cambridge, UK) and anti-CD163 (ab182422; Abcam, Cambridge, UK) polyclonal antibodies at a dilution of 1:500. Sections were subsequently processed for staining using PV-9000 two-step immunohistochemical staining kit (Zhongshan Jinqiao Biotechnology Company, Beijing, China) and 3,3-diaminobenzidin (DAB). Finally, sections were counterstained with hematoxylin and mounted. Negative controls were processed using normal rabbit serum (Dako, Carpinteria, CA, USA) as the primary antibody. Positive controls were performed using breast cancer tissue sections that had shown strong staining for the respective protein during the antibody optimization process.

### Evaluation of immunohistochemistry

**Fig. 1:**
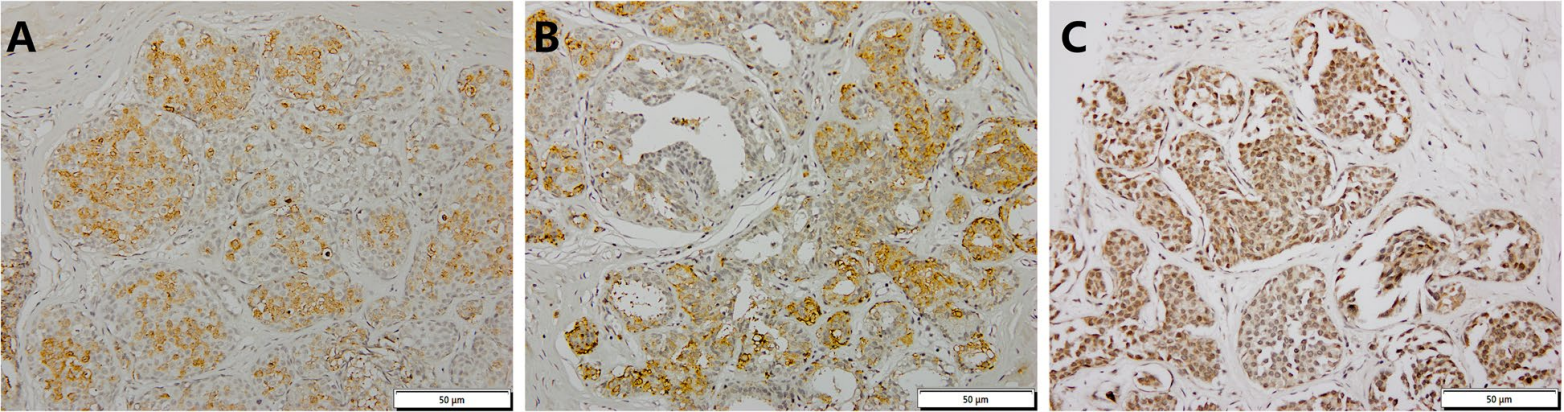
Representative staining images of CD68, CD163 and BMP2 in breast cancer tissues. **A**-**C** show the representative staining images of CD68, CD163 and BMP2, respectively

The immnunohistochemical staining results were evaluated independently in a blinded manner by two pathologists. Cases of disagreement were reviewed jointly to obtain a consensus score. The score of BMP-2 was evaluated by the staining extent multiplied by the staining intensity as described previously [5]. The expression of CD68 and CD163 were obtained by averaging the number of positively stained cells at high magnification (× 400) from five sampling areas. The representative staining images of CD68, CD163 and BMP-2 were shown in Fig. 1.

### Cell culture and induction of M2-like macrophage

Human breast cancer cell line MCF-7 (Shanghai cell bank, Shanghai, China) was cultured in Roswell Park Memorial Institure (RPMI)-1640 medium (Biological Industries, Beit Haemeq, Israel) containing 10% fetal calf serum (Biological Industries, Beit Haemeq, Israel) and 100 IU/ml penicillin (Biological Industries, Beit Haemeq, Israel).

Human leukemia monocyte THP-1 cells (Shanghai cell bank) were cultured in RPMI-1640 medium containing 100 ng/ml PMA (Sigma-Aldrich, St Louis, MO, USA) for 24 h to induce differentiation into the resting macrophages. The monocytes were further induced in M1-polarization medium containing 100 ng/ml LPS (Sigma-Aldrich,) and 20 ng/ml IFN-gamma (Sigma-Aldrich) for 48 h. Finally, M2-like macrophages were obtained by treatment with 20 ng/ml IL-4 (Sigma-Aldrich) for 48 h. The induced M2-like TAMs were confirmed by flow cytometry.

The induced M2-like TAMs were seeded on an insert for the subsequent co-culture with MCF-7 cells. The TAMs and MCF-7 cells were co-cultured without direct contact using a 6-well Transwell plate (0.4 μm) (Corning) for 48 h, and then washed for the following experiments. MCF-7 cells alone (without co-culture with TAMs) were used as a control.

### Alizarin red S staining and quantification of calcifications

The MCF-7 cells were fixed using 4% formaldehyde for 15 min after washing gently with PBS. Alizarin red S (ab146374, Abcam, Cambridge, UK) at the concentration of 0.01 g/ml (pH 7.5) was added to the fixed cells and incubated for 20 min. After alizarin red S solution was aspirated, the stained cells were washed and imaged using a camera mounted on a microscope. The Average Optical Density (AOD) of each well was obtained using the Image-J software.

### Western blot

After the TAMs and MCF-7 cells were co-cultured for two days, the MCF-7 cells were collected and lysed using RIPA lysis buffer containing PMSF. Following centrifugation, the supernatant was obtained to extract cellular proteins. Protein concentration was measured by BCA method. Electrophoresis was performed in an SDS-PAGE polyacrylamide gel and proteins were transferred to a PVDF membrane. The membrane was then incubated with rabbit anti-human BMP-2 at dilution of 1:1000 (ab214821, Abcam, Cambridge, UK) and GAPDH antibody (A19056, Abclonal, Wuhan, China) at dilution of 1:1000 as a loading control. After overnight incubation, the membrane was incubated with HRP-conjugated goat anti-rabbit secondary antibody (RS0002, immunoway, USA) at dilution of 1:1000. After washing, band was detected by placing the membrane in ECL luminescent solution and images were obtained by optical luminescence instrument. Relative protein levels were analyzed by using the Image-J software.

### Statistical analyses

Statistical analyses were carried out using SPSS v 19.0 and GraphPad Prism 8. Numerical variables were analyzed using t-test and continuous variables were analyzed using Pearson correlation coefficient. Categorical variables were analyzed using Chi-square test. Statistical significance of differential survival was assessed using the log-rank (score) test. Multivariate Cox regression analysis was performed for the expression of CD163, BMP-2, HER-2, axillary lymph node metastasis and microcalcifications. All *P* values presented were two-sided and the cutoff for significance was set at *P* ≤ 0.05.

## Results

### Characteristics of the study subjects

**Fig. 2:**
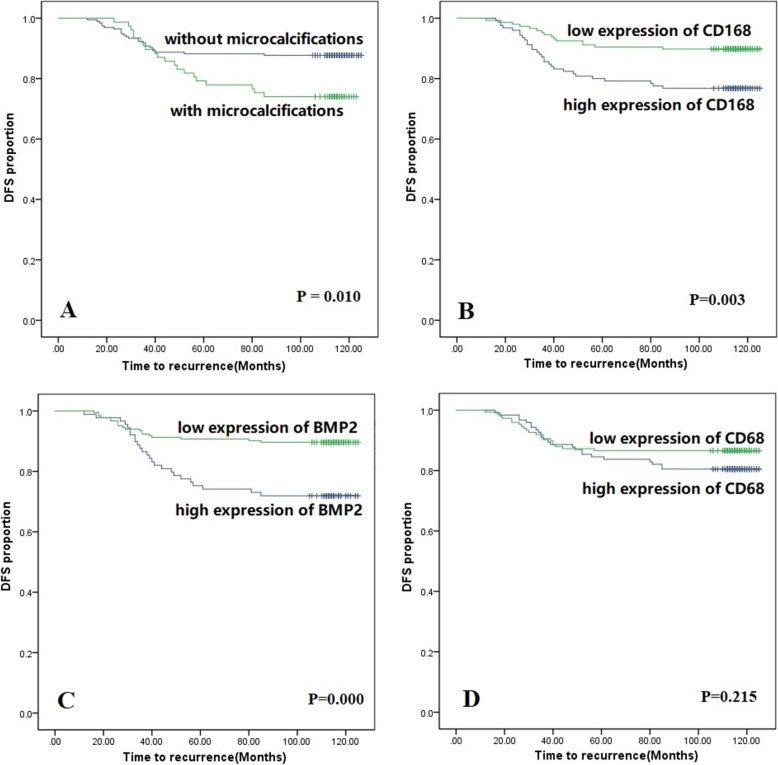
Relationship between microcalcifications or expression levels of CD68, CD163, BMP-2 and patients’ DFS. **A** shows that patients with microcalcifications were correlated with poor DFS (*p* = 0.010). **B** and **C** show that the high expressions of CD163 and BMP-2 were significantly correlated with poor DFS (*P* = 0.003, and 0.000, respectively). **D** shows Expression of CD68 was not correlated with prognosis with a *P* value of 0.215

The clinicopathological features of the 272 patients were shown in Table 1. The results showed that 28% (*n* = 77) of the patients had microcalcifications as detected by preoperative mammography and 49.6% (*n* = 135) had axillary metastasis (49.6%). Furthermore, 66.9% of patients (*n* = 182) had hormonal receptor-positive breast cancer and 20.2% (*n* = 55) patients had HER-2 receptor-positive breast cancer. Patients with microcalcifications were significantly correlated with HER-2 positivity (χ2 = 9.986, *P* = 0.002) and larger tumor size (χ2 = 9.629, *P* = 0.022) (Table 1). Patients with microcalcifications were significantly correlated with disease free survival (DFS) (χ2 = 6.645, *P* = 0.010) (Fig. 2A).

### High expression of CD163 and BMP-2 was significantly correlated with poor prognosis

CD68 and CD163 are the markers of the total macrophages and M2-like macrophages, respectively. Expression of CD68 and CD163 was found in cytoplasm and membrane. Positive staining of CD68 and CD163 was found in all sections. The median numbers of CD68-positive and CD163-positive cells were 30.6 (ranging from 6 to 77) and 21.7 (ranging from 4 to 63), respectively. We then graded CD68 and CD163 expression as either low or high according to the median number: the subjects with less than the median number was regarded as low expression group, and subjects with greater than the median number was regarded as high expression group. The expression of CD68 was significantly correlated with that of CD163 (correlation coefficient = 0.621, *P* = 0.000), indicating that most TAMs were M2-like macrophages. The staining of BMP-2 was found in cytoplasm, nucleus, and cell membrane. ROC curve analyses were used to dichotomize the expression scores of BMP-2 into high and low expression groups and the cutoff value was 7 which was obtained from the highest combined sensitivity and specificity at the end point of DFS.

Kaplan–Meier survival analyses were performed to assess the correlation of survival with the expressions of CD68, CD163, and BMP-2. The results showed that the high expression of CD163 (χ^2^ = 8.529, *P* = 0.003) and BMP-2 (χ^2^ = 13.296, *P* = 0.000) were significantly correlated with poor prognosis (Fig. 2B and Fig. 2C), but the expression of CD68 (χ^2^ = 1.538, *P* = 0.215) was not significantly correlated with prognosis (Fig. 2D). Patients were also classified into two subgroups based on the presence of microcalcifications. In the subgroup of patients with macrocalcifications, only high expression of BMP-2 was associated with poor prognosis (χ^2^ = 7.614; *P* = 0.006), while in the subgroup of patients without microcalcifications, only high expression of CD163 was associated with poor prognosis (χ^2^ = 6.412; *P* = 0.011).

### Infiltration of TAMs was significantly correlated with both microcalcifications and BMP-2 expression

Among the 123 patients with high expression of CD68, 46 patients (37.4%) had microcalcifications, while among the 149 patients with low expression of CD68, only 31 patients (20.8%) had microcalcifications. Similarly, among the 125 patients with high expression of CD163, 55 patients (44.0%) had microcalcifications, while among the 147 patients with low expression of CD163, only 22 patients (15.0%) had microcalcifications. Patients with high expression of CD68 and CD168 were more likely to be correlated with microcalcifications than those with low expression of CD68 and CD168 (χ^2^ = 9.141, 28.060 and *P* = 0.002, 0.000, respectively). Among the 89 patients with high expression of BMP-2, 49 patients (44.9%) had microcalcifications, while among the 183 patients with low expression of BMP-2, only 28 patients (15.3%) had microcalcifications. Patients with high expression of BMP-2 were more likely to be correlated with microcalcifications than those with low expression of BMP-2 (χ^2^ = 46.632, *P* = 0.000). The expression of CD68 and CD168 were also correlated with that of BMP-2 (correlation coefficient = 0.348 and 0.307, *P* = 0.000 and 0.000 respectively). Meanwhile, the expression of CD163 were also correlated with that of HER2 (correlation coefficient = 0.132, *P* = 0.029).

### Univariate analysis of correlation between other clinicopathological features and prognosis

Kaplan–Meier survival analyses were performed to determine the correlation of prognosis with hormonal receptor, HER-2, age, tumor size, axillary metastasis, surgical method and chemotherapy regimen. The results showed that HER-2 and axillary metastasis were risk factors of poor prognosis in breast cancer (χ^2^ = 4.586; *P* = 0.032 and χ^2^ = 12.383; *P* = 0.006, respectively). Other clinicopathological features had no significant predictive value for prognosis.

### Cox regression analysis

COX regression analysis was performed on the statistically significant variables in single factor analysis including the expression of CD163, BMP-2, HER-2, axillary lymph node metastasis and microcalcifications. BMP-2 and axillary lymph node metastasis were the only independent prognostic factors, with a hazard ratio of 2.155 (*P* = 0.023) and 1.426 (*P* = 0.011) respectively. Expression of CD163, HER-2 and microcalcifications was not independent prognostic factors for breast cancer (*P* = 0.078, 0.064 and 0.747 respectively).

### TAMs secrete BMP-2 and induce microcalcifications in breast cancer cells

**Fig. 3:**
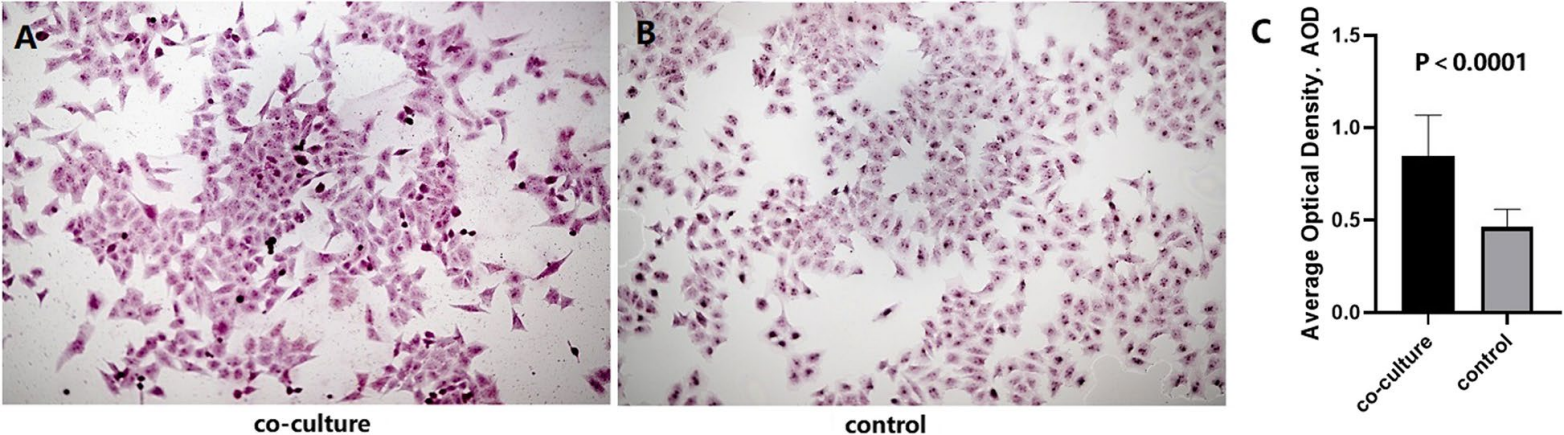
Representative images of alizarin red S staining and the average calcification content of the two group of the cells. **A** and **B** show the representative images of co-cultured and control cells stained with alizarin red S. **C** shows the bar plot comparison of AOD of the co-cultured and control cells. The average calcification content (AOD) of co-cultured cells is significantly increased compared to the control cells (*P*<0.0001)

**Fig. 4:**
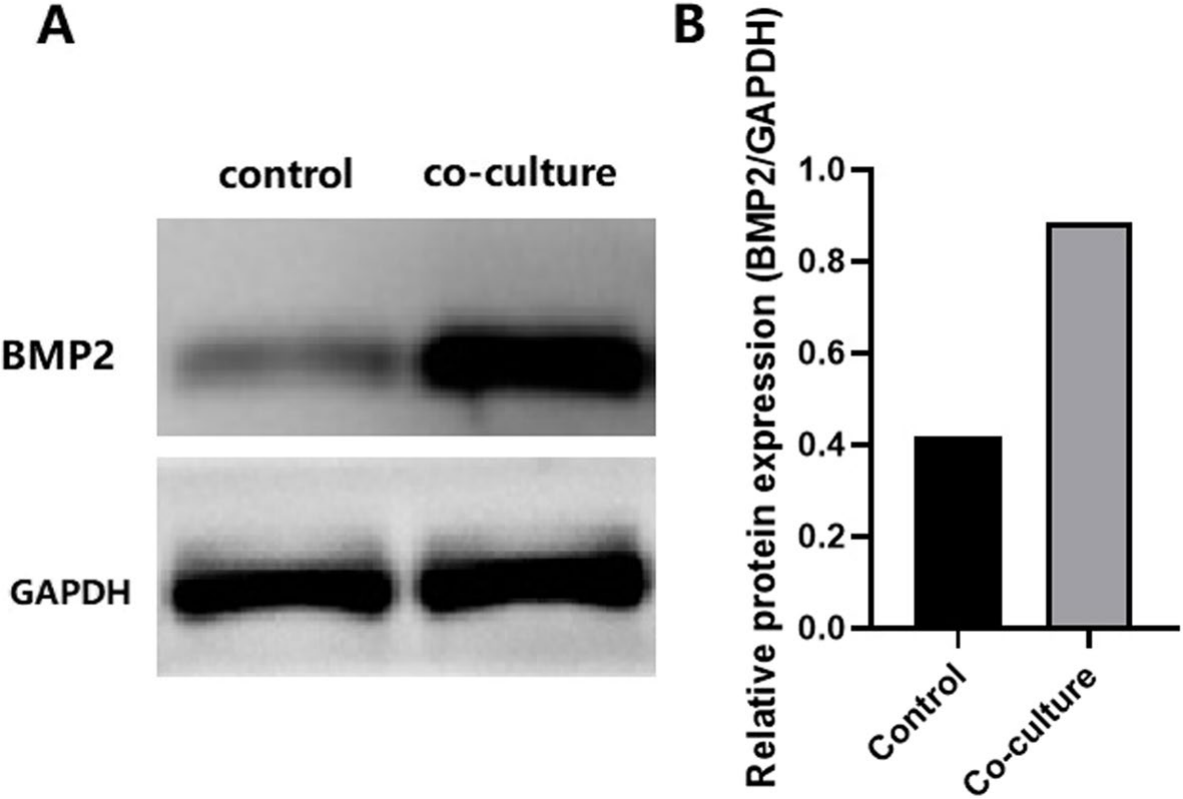
Western blot analysis of BMP2 protein in co-cultured and control cells. **A** shows the bands of BMP2 in co-cultured and control cells measured by Western blot analysis. **B** shows the bar plot comparison of relative protein expression values (gray value of BMP2/GAPDH). Full-length gels were shown in Supplementary Fig. S1 and Fig. S2

In order to further pinpoint the role of TAMs in microcalcifications of breast cancer cells, THP-1 cells were treated with PMA and IL-4 to induce differentiation into M2-like TAM which was verified by CD68 and CD163 expression (M2-like phenotype biomarker). Microcalcifications were determined in MCF-7 cells cultured with or without M2-like TAMs. The results showed that the formation of cellular microcalcifications is significantly increased in MCF-7 cells co-cultured with M2-like TAMs compared to MCF-7 cells alone (Fig. 3A and Fig. 3B). The average calcification content (Average Optical Density, AOD) of the cells was shown in Fig. 3C. Western blot results showed that BMP-2 expression was significantly upregulated in MCF-7 cells co-cultured with TAMs compared with MCF-7 cells alone (Fig. 4).

## Discussion

The mechanism underlying microcalcifications formation in breast cancer is still not fully understood. Previous studies have shown that BMP-2 expression was significantly correlated with the presence of microcalcifications [5] and BMP-2 can induce breast cancer cells to acquire osteoblastic characteristics [3, 4]. Recent studies also support the hypothesis that the process of microcalcifications is the crosstalk between the immune system and osteoclastogenesis [7]. BMP-2 overexpression has been shown to be associated with microcalcifications and is found to be produced by the tumor microenvironment, but not by the breast cancer cells themselves [29]. Macrophages, acting as both immune cells and osteoclast precursors, are one of the major immune cells in the tumor microenvironment [8, 31], and have been demonstrated to have the ability to secrete osteoinductive signals including BMP-2 [32], which plays important role in osteogenesis [31]. Recent study has shown that macrophages are correlated with microcalcifications in benign lesions and breast cancer cells may undergo osteoblast differentiation after co-culturing MDA-MB-231 with calcium oxalate and activate monocytes [33]. In this study, we showed that CD68 (a validated human pan-macrophage marker) and CD163 (a validated M2-like macrophage marker) [34, 35] were associated with microcalcifications in malignant lesions of breast, suggesting that TAMs play a role in breast microcalcifications deposition. This hypothesis was further supported by the results that the formation of cellular microcalcifications is significantly increased in MCF-7 cells that are co-cultured with TAMs. These results indicated that TAMs could be the cells that secrete BMP-2 and induce microcalcifications in breast cancer.

BMP signaling plays an important role in the development of embryonic mammary gland and maintaining tissue homeostasis [36, 37]. In cancer development, roles of BMPs signaling are more complex and can be cancer growth-promoting or inhibiting, though more recent studies have reported its oncogenic roles [3, 38, 39]. BMP-2 is also known to activate both canonical pathway (smad1/5/8) and noncanonical pathways (PI3K/AKT) to induce epithelial-mesenchymal transition (EMT) [39]. BMP-2 is overexpressed in bone metastases compared to metastases from other sites [40]. Several studies have demonstrated that BMP-2 could also upregulate some bone metabolic factor, e.g., RANKL and RUNX2, to induce breast cancer cells acquire osteoblastic characteristics. Thus, the term breast osteoblast-like cells (BOLCs) was introduced by Scimeca et al. [3, 41–43]. BOLCs could both produce the microcalcifications and promotes EMT and tumor bone metastasis [44–46]. Meanwhile, our previous study also showed that the BMP-2 may lead to the upregulation of the AKT/mTOR pathway, another potential contributor to poor prognosis. These results are consistent with our finding that BMP-2 correlates with poor prognosis and this may be due to the activation of its downstream signals. A recent study also showed that a nuclear variant of BMP-2 [47] is more strongly correlated with microcalcifications and the cytoplasmic variant BMP-2 is more correlated with EMT [3], but the antibody of the variants of BMP-2 was the same. In the subgroup analysis, BMP-2 was found to be only correlated with poor prognosis in patients with microcalcifications, which may be due to the different roles of BMP-2 variants.

Numerous studies have shown that TAMs are capable of affecting breast cancer cells in a variety of aspects: including tumor growth, metastasis, therapy resistance, and adverse clinicopathological characteristics such as larger tumor size, lymph node metastasis, HR negativity, and HER2 expression [15]. This is consistent with our results showing a significant correlation between TAMs (CD163) and microcalcifications and HER2. Previous studies have also shown that the presence of microcalcifications is correlated with HER2 [27, 48, 49], which may also be partially due to the effect of TAMs. TAMs were also shown to mediate the anti-HER2 targeted treatment and removal of TAMs could significantly increase the therapeutic effect of anti-HER2 [50]. This indicates that breast cancer patients with microcalcifications might be more likely to show resistance towards anti-HER2 agents and may benefit from additional TAMs targeting therapy. We also found that the M2-like macrophages (expression of CD163) were significantly correlated with poor prognosis, which is also consistent with previous studies [12, 18, 51, 52]. However, it is not an independent prognostic factor in our study. M2-like macrophages were the only factor found to be significantly associated with poor prognosis in patients without microcalcifications. This may be due to the small sample size or existence of other factors that affect prognosis. Conversely, the pan-macrophage marker, CD68, was not associated with prognosis in any group, which may be due to the anti-tumor role of M1-like macrophages.

## Conclusions

In summary, the results from this study support the proposed hypothesis that TAMs could secrete BMP-2 to induce microcalcifications in breast cancer and may influence prognosis via multiple pathways including BMP-2 and its downstream factors. Further studies are needed to elucidate the mechanisms by which BMP-2 induces microcalcifications deposition and its role in biological behavior of tumors.

## Supplementary Information

**Additional file 1: Figure S1.**

**Additional file 2: Figure S2.**

## Data Availability

The data used to support the findings of this study are available from the corresponding author upon request.

## References

[1] Marmot MG, Altman DG, Cameron DA, Dewar JA, Thompson SG, Wilcox M (2013). The benefits and harms of breast cancer screening: an independent review. Br J Cancer.

[2] Bent CK, Bassett LW, D'Orsi CJ, Sayre JW (2010). The positive predictive value of BI-RADS microcalcification descriptors and final assessment categories. AJR Am J Roentgenol.

[3] Scimeca M, Giocondo R, Montanaro M, Granaglia A, Bonfiglio R, Tancredi V, Mauriello A, Urbano N, Schillaci O, Bonanno E (2020). BMP-2 variants in breast epithelial to mesenchymal transition and microcalcifications origin. Cells.

[4] Scimeca M, Giannini E, Antonacci C, Pistolese CA, Spagnoli LG, Bonanno E (2014). Microcalcifications in breast cancer: an active phenomenon mediated by epithelial cells with mesenchymal characteristics. BMC Cancer.

[5] Wang S, Gu M, Jiang H, Zheng X (2020). BMP-2 upregulates the AKT/mTOR pathway in breast cancer with microcalcification and indicates a poor prognosis. Clin Transl Oncol.

[6] Sharma T, Radosevich JA, Pachori G, Mandal CC (2016). A molecular view of pathological microcalcification in breast Cancer. J Mammary Gland Biol Neoplasia.

[7] Clemenceau A, Michou L, Diorio C, Durocher F (2020). Breast Cancer and microcalcifications: an Osteoimmunological disorder?. Int J Mol Sci.

[8] Coussens LM, Werb Z (2002). Inflammation and cancer. Nature.

[9] Yam M, Tchou J, English R, Highnam R, Highnam R, Roskell D, Greenall M, Brady M (2001). A mammographic dilemma: calcification or haemosiderin as a cause of opacities? Validation of a new digital diagnostic tool. Br J Radiol.

[10] Biswas SK, Allavena P, Mantovani A (2013). Tumor-associated macrophages: functional diversity, clinical significance, and open questions. Semin Immunopathol.

[11] Hussein MR, Hassan HI (2006). Analysis of the mononuclear inflammatory cell infiltrate in the normal breast, benign proliferative breast disease, in situ and infiltrating ductal breast carcinomas: preliminary observations. J Clin Pathol.

[12] Tiainen S, Tumelius R, Rilla K, Hamalainen K, Tammi M, Tammi R, Kosma VM, Oikari S, Auvinen P (2015). High numbers of macrophages, especially M2-like (CD163-positive), correlate with hyaluronan accumulation and poor outcome in breast cancer. Histopathology.

[13] Mahmoud SMA, Lee AHS, Paish EC, Macmillan RD, Ellis IO, Green AR (2012). Tumour-infiltrating macrophages and clinical outcome in breast cancer. J Clin Pathol.

[14] Murri AM, Hilmy M, Bell J, Wilson C, McNicol AM, Lannigan A, Doughty JC, McMillan DC (2008). The relationship between the systemic inflammatory response, tumour proliferative activity, T-lymphocytic and macrophage infiltration, microvessel density and survival in patients with primary operable breast cancer. Br J Cancer.

[15] Qiu SQ, Waaijer SJH, Zwager MC, de Vries EGE, van der Vegt B, Schroder CP (2018). Tumor-associated macrophages in breast cancer: innocent bystander or important player?. Cancer Treat Rev.

[16] Noy R, Pollard JW (2014). Tumor-associated macrophages: from mechanisms to therapy. Immunity.

[17] Mantovani A, Sozzani S, Locati M, Allavena P, Sica A (2002). Macrophage polarization: tumor-associated macrophages as a paradigm for polarized M2 mononuclear phagocytes. Trends Immunol.

[18] Sousa S, Brion R, Lintunen M, Kronqvist P, Sandholm J, Monkkonen J, Kellokumpu-Lehtinen PL, Lauttia S, Tynninen O, Joensuu H (2015). Human breast cancer cells educate macrophages toward the M2 activation status. Breast Cancer Res.

[19] Holness CL, Simmons DL (1993). Molecular cloning of CD68, a human macrophage marker related to lysosomal glycoproteins. Blood.

[20] Tang X (2013). Tumor-associated macrophages as potential diagnostic and prognostic biomarkers in breast cancer. Cancer Lett.

[21] Tabár L, Chen H-H, Duffy SW, Yen MF, Chiang CF, Dean PB, Smith RA (2000). A novel method for prediction of long-term outcome of women with T1a, T1b, and 10–14 mm invasive breast cancers: a prospective study. Lancet.

[22] Thurfjell E, Thurfjell MG, Lindgren A (2001). Mammographic finding as predictor of survival in 1-9 mm invasive breast cancers. Worse prognosis for cases presenting as calcifications alone. Breast Cancer Res Treat.

[23] Gajdos C, Tartter PI, Bleiweiss IJ, Hermann G, de Csepel J, Estabrook A, Rademaker AW (2002). Mammographic appearance of nonpalpable breast cancer reflects pathologic characteristics. Ann Surg.

[24] Zunzunegui RG, Chung MA, Oruwari J, Golding D, Marchant DJ, Cady B (2003). Casting-type calcifications with invasion and high-grade ductal carcinoma in situ: a more aggressive disease?. Arch Surg.

[25] Bennett RL, Evans AJ, Kutt E, Record C, Bobrow LG, Ellis IO, Hanby A, Moss SM (2011). Pathological and mammographic prognostic factors for screen detected cancers in a multi-Centre randomised, controlled trial of mammographic screening in women from age 40 to 48 years. Breast.

[26] Holmberg L, Wong YN, Tabar L, Ringberg A, Karlsson P, Arnesson LG, Sandelin K, Anderson H, Garmo H, Emdin S (2013). Mammography casting-type calcification and risk of local recurrence in DCIS: analyses from a randomised study. Br J Cancer.

[27] Ling H, Liu ZB, Xu LH, Xu XL, Liu GY, Shao ZM (2013). Malignant calcification is an important unfavorable prognostic factor in primary invasive breast cancer. Asia Pac J Clin Oncol.

[28] Tabar L, Tony Chen HH, Amy Yen MF, Tot T, Tung TH, Chen LS, Chiu YH, Duffy SW, Smith RA (2004). Mammographic tumor features can predict long-term outcomes reliably in women with 1-14-mm invasive breast carcinoma. Cancer.

[29] Chapellier M, Bachelard-Cascales E, Schmidt X, Clement F, Treilleux I, Delay E, Jammot A, Menetrier-Caux C, Pochon G, Besancon R (2015). Disequilibrium of BMP2 levels in the breast stem cell niche launches epithelial transformation by overamplifying BMPR1B cell response. Stem Cell Rep.

[30] Dube PR, Birnbaumer L, Vazquez G (2017). Evidence for constitutive bone morphogenetic protein-2 secretion by M1 macrophages: constitutive auto/paracrine osteogenic signaling by BMP-2 in M1 macrophages. Biochem Biophys Res Commun.

[31] Wei F, Zhou Y, Wang J, Liu C, Xiao Y (2018). The immunomodulatory role of BMP-2 on macrophages to accelerate osteogenesis. Tissue Eng A.

[32] Champagne CM, Takebe J, Offenbacher S, Cooper LF (2002). Macrophage cell lines produce osteoinductive signals that include bone morphogenetic protein-2. Bone.

[33] Scimeca M, Bonfiglio R, Menichini E, Albonici L, Urbano N, De Caro MT, Mauriello A, Schillaci O, Gambacurta A, Bonanno E (2019). Microcalcifications drive breast Cancer occurrence and development by macrophage-mediated epithelial to mesenchymal transition. Int J Mol Sci.

[34] Gottfried E, Kunz-Schughart LA, Weber A, Rehli M, Peuker A, Müller A, Kastenberger M, Brockhoff G, Andreesen R, Kreutz M (2008). Expression of CD68 in non-myeloid cell types. Scand J Immunol.

[35] Ambarus CA, Krausz S, van Eijk M, Hamann J, Radstake TR, Reedquist KA, Tak PP, Baeten DL (2012). Systematic validation of specific phenotypic markers for in vitro polarized human macrophages. J Immunol Methods.

[36] Ren J, ten Dijke P (2017). Bone morphogenetic proteins in the initiation and progression of breast Cancer. Bone Morphogenetic Proteins: Systems Biology Regulators edn.

[37] Wang RN, Green J, Wang Z, Deng Y, Qiao M, Peabody M, Zhang Q, Ye J, Yan Z, Denduluri S (2014). Bone morphogenetic protein (BMP) signaling in development and human diseases. Genes Dis.

[38] Frey P, Devisme A, Schrempp M, Andrieux G, Boerries M, Hecht A (2020). Canonical BMP signaling executes epithelial-mesenchymal transition downstream of SNAIL1. Cancers (Basel).

[39] Huang P, Chen A, He W, Li Z, Zhang G, Liu Z, Liu G, Liu X, He S, Xiao G (2017). BMP-2 induces EMT and breast cancer stemness through Rb and CD44. Cell death discovery.

[40] Zhang XH, Wang Q, Gerald W, Hudis CA, Norton L, Smid M, Foekens JA, Massague J (2009). Latent bone metastasis in breast cancer tied to Src-dependent survival signals. Cancer Cell.

[41] Bonfiglio R, Granaglia A, Giocondo R, Scimeca M, Bonanno E (2020). Molecular aspects and prognostic significance of microcalcifications in human pathology: a narrative review. Int J Mol Sci.

[42] Scimeca M, Urbano N, Bonfiglio R, Schillaci O, Bonanno E (2018). Breast osteoblast-like cells: a new biomarker for the management of breast cancer. Br J Cancer.

[43] Bonfiglio R, Scimeca M, Urbano N, Bonanno E, Schillaci O (2018). Breast microcalcifications: biological and diagnostic perspectives. Future Oncol.

[44] Scimeca M, Antonacci C, Toschi N, Giannini E, Bonfiglio R, Buonomo CO, Pistolese CA, Tarantino U, Bonanno E (2018). Breast osteoblast-like cells: a reliable early marker for bone metastases from breast Cancer. Clin Breast Cancer.

[45] Scimeca M, Trivigno D, Bonfiglio R, Ciuffa S, Urbano N, Schillaci O, Bonanno E (2021). Breast cancer metastasis to bone: from epithelial to mesenchymal transition to breast osteoblast-like cells. Semin Cancer Biol.

[46] Kim B, Kim H, Jung S, Moon A, Noh DY, Lee ZH, Kim HJ, Kim HH (2020). A CTGF-RUNX2-RANKL Axis in breast and prostate Cancer cells promotes tumor progression in bone. J Bone Miner Res.

[47] Tellez Freitas CM, Burrell HR, Valdoz JC, Hamblin GJ, Raymond CM, Cox TD, Johnson DK, Andersen JL, Weber KS, Bridgewater LC (2019). The nuclear variant of bone morphogenetic protein 2 (nBMP2) is expressed in macrophages and alters calcium response. Sci Rep.

[48] Sun SS, Zhang B, Zhao HM, Cao XC (2014). Association between mammographic features and clinicopathological characteristics in invasive ductal carcinoma of breast cancer. Mol Clin Oncol.

[49] Nie Z, Wang J, Ji XC. Microcalcification-associated breast cancer: HER2-enriched molecular subtype is associated with mammographic features. Br J Radiol. 2018;20170942. 10.1259/bjr.20170942 29927639

[50] Xu M, Liu M, Du X, Li S, Li H, Li X, Li Y, Wang Y, Qin Z, Fu YX (2015). Intratumoral delivery of IL-21 overcomes anti-Her2/Neu resistance through shifting tumor-associated Macrophages from M2 to M1 phenotype. J Immunol.

[51] Zhang WJ, Wang XH, Gao ST, Chen C, Xu XY, Sun Q, Zhou ZH, Wu GZ, Yu Q, Xu G (2018). Tumor-associated macrophages correlate with phenomenon of epithelial-mesenchymal transition and contribute to poor prognosis in triple-negative breast cancer patients. J Surg Res.

[52] Klingen TA, Chen Y, Aas H, Wik E, Akslen LA (2017). Tumor-associated macrophages are strongly related to vascular invasion, non-luminal subtypes, and interval breast cancer. Hum Pathol.

