# Supplementary figures and images for: Secretion of BMP-2 by tumor-associated macrophages (TAM) promotes microcalcifications in breast cancer
Source: BMC Cancer. 2022 Jan 4;22:34. doi: 10.1186/s12885-021-09150-3 (PMC8729115; doi:10.1186/s12885-021-09150-3)

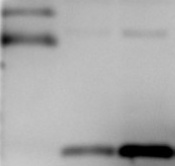

Supplement: Supplementary file 1 — Additional file 1: Figure S1. [file 12885_2021_9150_MOESM1_ESM.tif]

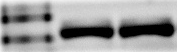

Supplement: Supplementary file 2 — Additional file 2: Figure S2. [file 12885_2021_9150_MOESM2_ESM.tif]
